# Pursuing Better Outcomes in Obstructive Colorectal Cancer Surgery: A New Predictive Scoring System for Immediate Complications and Optimization of Hospital Stay

**DOI:** 10.7759/cureus.76237

**Published:** 2024-12-23

**Authors:** Alexandra-Ana Mihailescu, Serban Dragosloveanu, Minodora Onisâi, Matei Teodorescu, Adrian Alexandru, Catalin Alius, Corneliu-Dan Blendea, Stefan-Ilie Neagu, Dragos Serban, Sebastian Gradinaru

**Affiliations:** 1 Faculty of Medicine, Carol Davila University of Medicine and Pharmacy, Bucharest, ROU; 2 Department of Anesthesiology and Intensive Care, Foisor Hospital Bucharest, Bucharest, ROU; 3 Department of Orthopedics, Foisor Hospital Bucharest, Bucharest, ROU; 4 Department of Hematology, Emergency University Hospital Bucharest, Bucharest, ROU; 5 Neurological Recovery Clinic, Elias University Emergency Hospital, Bucharest, ROU; 6 Emergency University Hospital Bucharest, Department of Plastic and Reconstructive Surgery, Bucharest, ROU; 7 Emergency University Hospital Bucharest, 4th Surgery Department, Bucharest, ROU; 8 Department of Medical-Clinical Disciplines, General Surgery, Titu Maiorescu University of Bucharest, Bucharest, ROU; 9 Ilfov County Emergency Clinical Hospital, Department of Recovery, Physical Medicine and Balneology, Bucharest, ROU; 10 Ilfov County Emergency Clinical Hospital, Department of General Surgery, Bucharest, ROU

**Keywords:** colorectal cancer, complication score, estimated discharge day, hospital stay, intestinal obstruction

## Abstract

Introduction: Colorectal cancer (CRC) is one of the most common cancers occurring globally. Surgery for CRC often extends hospital stays due to complications, as patients must meet nutritional needs and regain mobility before discharge. Longer hospital stays, required for extended monitoring and care, can increase the risk of further complications, creating a cycle where extended stays lead to more issues. Predicting a patient’s length of stay (LOS) is crucial for optimal resource management, financial control, and patient care.

Methods: This study aimed to create a scoring system to predict postoperative complications and prolonged hospitalization in colorectal cancer surgery patients. Over 60 variables, including age, BMI, and tumor location, were analyzed for their correlation with complications.

Results: A complication score was developed based on six factors linked to postoperative complications: hemoglobin (Hb), serum albumin, tumor localization, EC (epidural catheter), opioid use, and NPO (nil per os) days. Patients with three or more identified risk factors had a 6.17-fold higher complication rate, with a highly significant p-value of 0.0008, demonstrating the score's strong potential for identifying high-risk patients. The factors significantly associated with length of stay (LOS) include admission hemoglobin levels, tumor localization (right versus left colon), intraoperative fluid intake, the presence or absence of regional anesthesia and analgesia (RAA), the number of drainage tubes, and postoperative hematocrit levels. The analysis shows that patients with at least three of the six identified risk factors are 5.17 times more likely to experience prolonged hospitalization (over eight days) compared to those with fewer than three points, with a statistically significant correlation (p-value of 0.003). Our findings indicate that patients with three or more risk factors are significantly more likely to experience complications and extended hospital stays.

Conclusions: This scoring system can serve as an essential tool for healthcare providers to identify at-risk patients, optimize resource allocation, and ultimately enhance patient recovery and outcomes. Moreover, the integration of the complication and LOS scores into routine preoperative assessments can facilitate a more personalized care plan, enabling healthcare providers to identify patients who may benefit from closer monitoring and additional support during their recovery. Further validation in diverse populations and settings is needed to confirm the scoring system’s generalizability and utility.

## Introduction

Colorectal cancer (CRC) is the third most prevalent cancer globally, with an average 5-year overall survival rate of 60% [1]. CRC surgery is conducted extensively worldwide to address both benign and malignant conditions. Given the nature of colorectal cancer surgery (CRS), patients generally remain hospitalized until they can effectively manage their nutritional needs and regain the ability to walk independently [2].

The relationship between complications and length of stay (LOS) in a hospital is dynamic and interconnected. When complications arise during a patient's treatment, they often lead to an extended hospital stay [3-8]. This is because complications, whether they are infections, surgical issues, or other medical setbacks, require additional time for management, treatment, and recovery [9-12]. Extended monitoring, more intensive care, and possibly further interventions are needed, all of which contribute to a longer stay. Conversely, a prolonged hospital stay itself can increase the risk of complications [2, 13]. This bidirectional relationship creates a challenging cycle. Complications prolong the hospital stay, and the longer the stay, the greater the risk of additional complications [14-18].

Accurately predicting a patient’s LOS is an aspect that significantly influences various dimensions of hospital operations and patient care. Accurate forecasts allow healthcare facilities to allocate resources such as beds, medical staff, and equipment more effectively. Financial management and cost control are other key areas impacted by LOS predictions. Moreover, predicting LOS contributes to improving patient care and enhancing the overall hospital experience [2,19]. Colorectal resections typically result in an average hospital stay ranging from 6 to 11 days and carry a complication rate between 15% and 20% [20].

The ability to accurately predict postoperative complications is critical for optimizing patient care, improving outcomes, enhancing patient safety, and managing healthcare resources efficiently. It is a key component of modern surgical practice that drives both clinical and operational success. The aim of this study was to develop a scoring system designed to offer healthcare professionals a predictive tool that assesses the likelihood of postoperative complications and the potential for prolonged hospitalization in patients who have undergone colorectal cancer surgery.

Over 60 variables were analyzed, including age, sex, BMI, the interval between admission and surgery, days of hospitalization, Hb at admission, days without preoperative transit, type of analgesia, type of anesthesia, tumor location, and complications. Among these, some were found to be positively or negatively correlated with the occurrence of complications. Six parameters are considered in calculating the complication score, which represents the risk factors for complications: hemoglobin (Hb), preoperative serum albumin, tumor localization, EC (epidural catheter), use of opioids, and NPO (nil per os).

Significant factors associated with increased hospitalization across the entire cohort included opioid use, NPO days, and fluid intake in the first 3 days. These factors are part of the ERAS (enhanced recovery after surgery) protocol, which might introduce biases into the statistical research objective of increasing hospitalization duration, as they are directly correlated with it. Since reducing hospitalization duration is a primary goal of ERAS, these parameters were not be included in the ERAS criteria. Statistically significant differences in hospitalization duration (0-7 days vs. 8+ days) were observed with respect to the following factors: hemoglobin levels at admission (Hb), right versus left colon, fluid intake intraoperatively, presence or absence of regional anesthesia and analgesia (RAA), number of drainage tubes, and postoperative hematocrit (Ht) level.

## Materials and methods

Identification of patients

Data was collected from 120 consecutive patients treated for obstructive colorectal cancer at an emergency hospital. The patients presented for emergency care. This retrospective study focused on a cohort of individuals who received treatment between January 2022 and December 2023. Data was collected using electronic health records.

Criteria of exclusion

Patients presenting with clinical peritonitis, those with tumors exhibiting recurrent obstruction, and individuals undergoing neoadjuvant therapy were excluded from the study. Additionally, patients with non-resectable tumors or those diagnosed with metastatic disease were not included in the analysis. From an initial pool of 167 patients, 47 individuals were excluded after applying the predefined exclusion criteria, leaving a final cohort of 120 patients for analysis.

Statistical analysis 

Statistical analysis was conducted using IBM Statistical Package for the Social Sciences (SPSS) version 25 (IBM Corp., Armonk, NY, USA), Epi Info Version 7.2 (Center for Disease Control and Prevention, Atlanta, GA, USA), and Microsoft Excel from the Microsoft 365 suite (Microsoft Corp., Redmond, WA, USA). Categorical variables were presented as numbers and percentages. The Mann-Whitney U test was used to assess differences between independent groups. For small sample sizes, we applied the mid-p method and Fisher Exact tests. The risk score for complications was developed using receiver operating characteristic (ROC) curve analysis and odds ratio. ROC curve analysis was used to identify the optimal cut-off points for numerical values within our group. Based on the identified cut-off values, we dichotomized the variables. The odds ratio was used to quantify the association between the dichotomized variables and the outcome. A statistical significance level of p<0.05 was considered for all analyses.

## Results

Complication score

**Figure 1:**
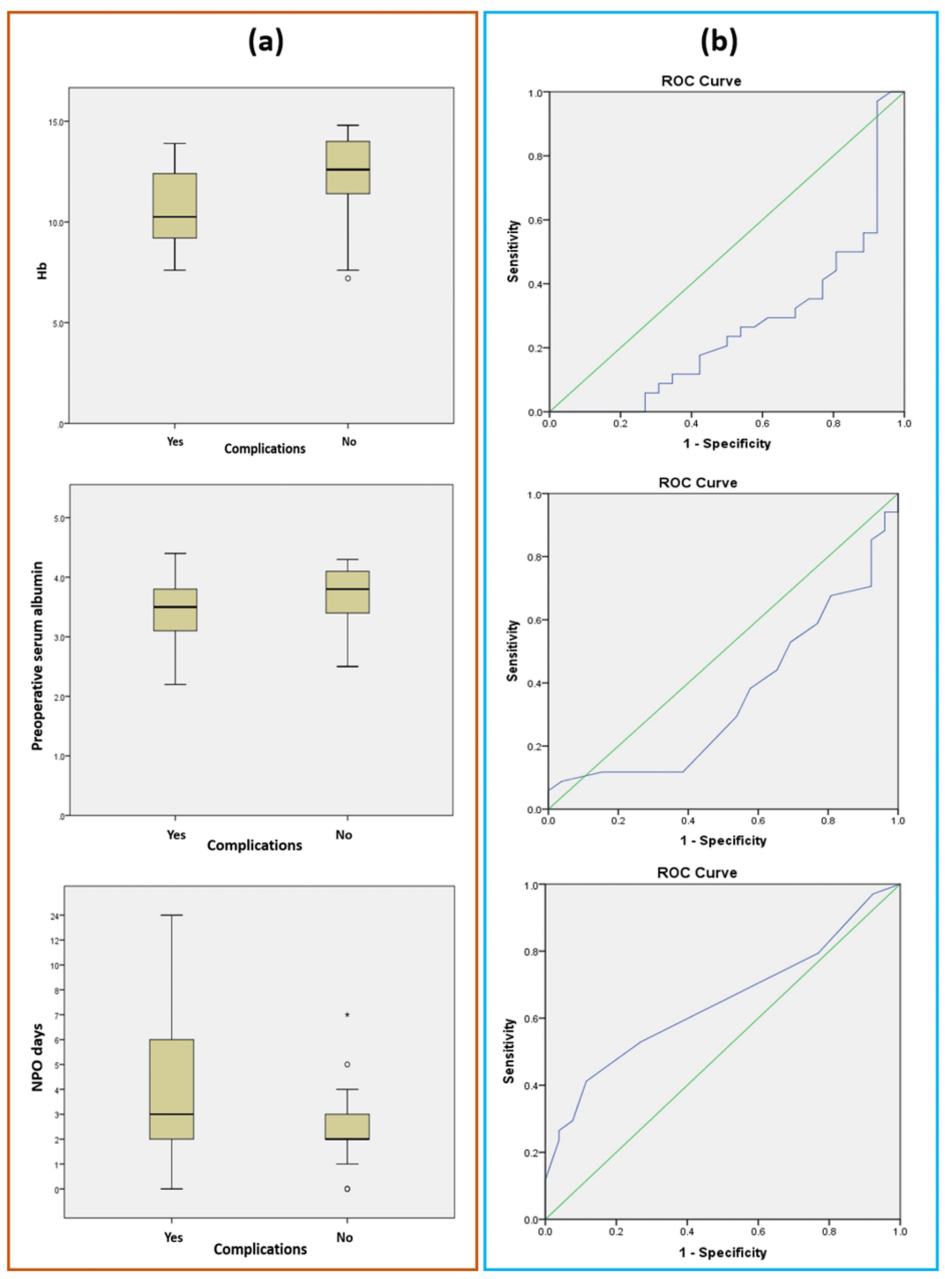
Relationship between Hb (hemoglobin) levels, preoperative serum albumin, NPO (nill per os) duration, and the incidence of postoperative complications. (a) Boxplot diagram comparing the distribution of patient groups based on the presence or absence of postoperative complications (mean±SD); (b) The ROC curve evaluates the predictive accuracy of variables (Hb, preoperative serum albumin, NPO) in identifying patients at higher risk for postoperative complications, with the area under the curve (AUC), reflecting their overall discriminative power.

The score for complications is calculated based on six key parameters that indicate the risk factors for complications: hemoglobin (Hb) levels, preoperative serum albumin, tumor localization, the presence of an epidural catheter (EC), opioid use, and the duration of NPO (nil per os) status. Hemoglobin (Hb) levels represent hemoglobin measured upon admission. Preoperative serum albumin represents the level of albumin measured before the surgical procedure. Tumor localization is a parameter that identifies whether the tumor is situated in the right-sided colon (including the cecum, ascending colon, and proximal transverse colon) or the left-sided colon (including the distal transverse colon, descending colon, sigmoid colon, and rectum). The Epidural Catheter (EC) parameter records whether an epidural catheter was utilized during the procedure, indicated as "yes" when used and "no" when not used. The opioid use parameter indicates whether opioids were administered. The parameter was recorded as "yes" if opioids were used and "no" if they were not. NPO status refers to the period after surgery during which a patient is not allowed to consume any food, liquids, or oral medications. Figure 1 illustrates the relationship between Hb levels, preoperative serum albumin, and NPO duration in relation to the incidence of postoperative complications. Panel (a) presents a boxplot diagram that visually compares the distribution of these parameters among patients with and without postoperative complications, highlighting significant variations across these factors. Panel (b) displays the ROC curve, evaluating the predictive accuracy of these variables in identifying patients at higher risk for postoperative complications, with the area under the curve (AUC), reflecting their overall discriminative power.

It was found that there is a strong relationship between tumor localization and the risk of complications, with a p-value of 0.0190. A very strong correlation between the use of an epidural catheter and the risk of complications was also found with a p-value of 0.07207. Also the association between opioid use and the risk of complications is also highly statistically significant, with a p-value of 0.02138.

**Figure 2:**
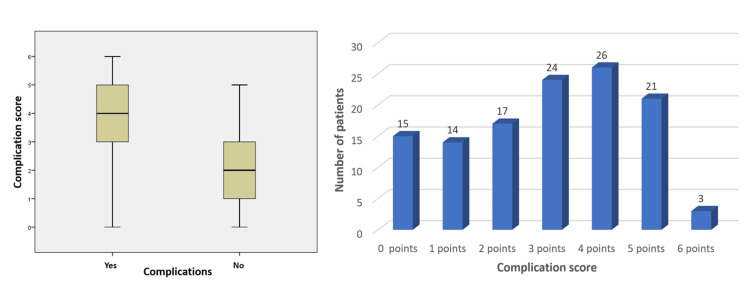
Graphical representation of the relationship between postoperative complications and complication scores, indicating that higher complication scores are linked to an increased incidence of complications.

For the complication score, we assigned each patient 1 point for each of the factors that have been proven to be significantly associated with the development of postoperative complications: Hb<11.05 g/dL; preoperative serum albumin<3.55 g/dL; tumor localization: right-sided colon; EC: NO; use of opioids: yes; NPO (nothing by mouth) duration>2.5 days. Thus, patients who accumulated at least three out of the six possible points on this complication score had a complication rate that was 6.17 times higher compared to those with fewer points. This finding is highly statistically significant, with a p-value of 0.0008, indicating a very strong correlation between the score and the likelihood of postoperative complications. The high significance of this result suggests that the score is a reliable predictor of complications, underscoring its potential value in clinical practice for identifying high-risk patients. Figure 2 shows that higher complication scores are associated with a higher incidence of complications. This representation helps to emphasize the predictive power of the complication score in assessing postoperative risk.

LOS score

Statistically significant differences in hospitalization duration, categorized as 0-7 days versus 8+ days, were observed across several key factors. These include Hb levels at the time of admission, tumor localization (right-sided vs. left-sided colon), the volume of intraoperative fluid intake, the use or absence of regional anesthesia and analgesia (RAA), the number of drainage tubes placed, and postoperative Ht levels. Each variable demonstrated a meaningful impact on the length of stay, suggesting their importance in predicting and managing patient recovery time after surgery. Hb levels at the time of admission represent hemoglobin measured upon admission. Tumor localization is a parameter that identifies whether the tumor is situated in the right-sided colon (including the cecum, ascending colon, and proximal transverse colon) or the left-sided colon (including the distal transverse colon, descending colon, sigmoid colon, and rectum). The volume of intraoperative fluid intake is a parameter that refers to the total amount of fluids (such as intravenous fluids, blood products, and other solutions) administered to a patient during a surgical procedure. The use of the RAA parameter indicates whether regional anesthesia and analgesia techniques were employed during the procedure, which is recorded as "yes" if utilized and "no" if not. The number of drainage tubes placed refers to the total number of tubes inserted into a patient's body after surgery. Postoperative hematocrit (Ht) levels refer to the percentage of red blood cells in a patient's blood measured after surgery.

**Figure 3:**
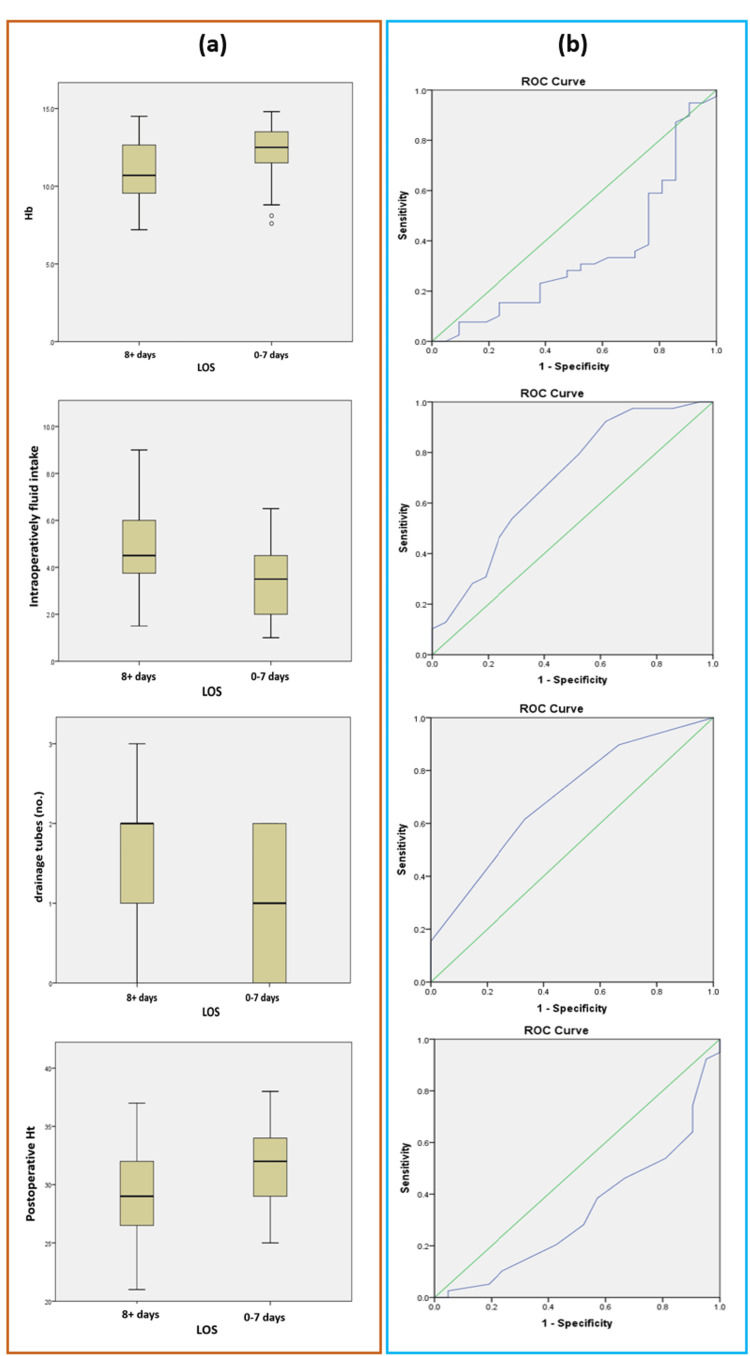
Relationship between Hb (hemoglobin) levels at admission, intraoperatively fluid intake, number of drainage tubes, postoperative Ht (hematocrit) level and hospital stay. (a) Boxplot diagram comparing the distribution of patient groups based on the hospital stay duration categorized as either 0-7 days or 8+ days (mean±SD); (b) The ROC curve evaluates the predictive accuracy of variables (Hb levels at admission, intraoperatively fluid intake, number of drainage tubes, postoperative Ht level) in identifying patients at higher risk for postoperative complications, with the area under the curve (AUC), reflecting their overall discriminative power.

Figure 3 illustrates the relationship between Hb levels at admission, intraoperative fluid intake, number of drainage tubes, postoperative hematocrit levels, and length of hospital stay categorized as either 0-7 days or 8+ days. Panel (a) features a boxplot diagram showing the distribution of these factors in relation to the two hospital stay groups. Panel (b) displays the ROC curve, evaluating the predictive ability of these variables for extended hospital stays (8+ days), with the area under the curve indicating their effectiveness in distinguishing between shorter and longer stays.

**Figure 4:**
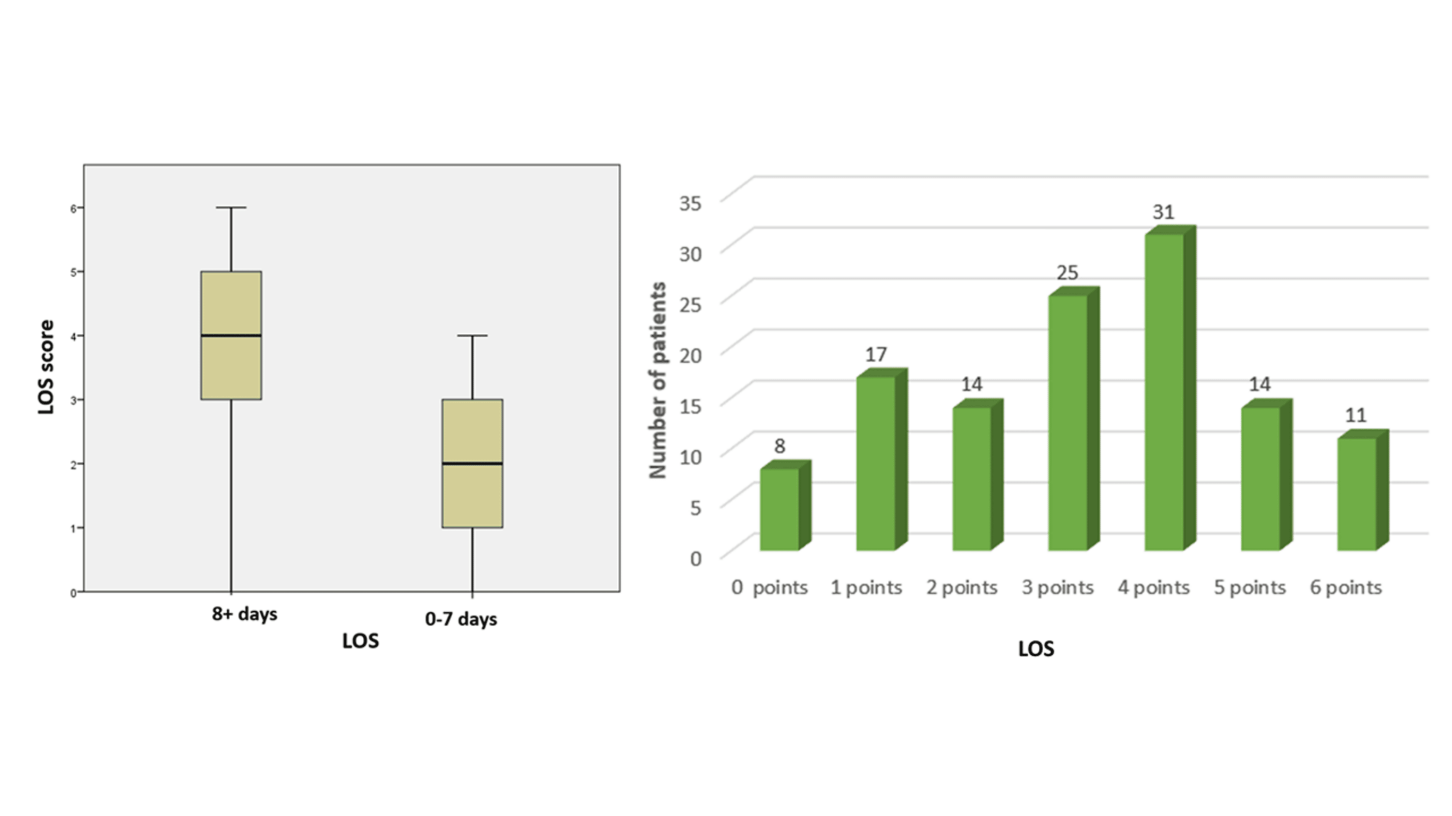
Graph illustrating the relationship between hospital stay and LOS score showing that higher LOS scores are associated with longer hospital stays.

It was found that there is a strong relationship between tumor localization (right/left colon) and LOS, with a p-value of 0.0492. Also, the association between the presence or absence of RAA and LOS is also highly statistically significant, with a p-value of 0.0236. For the risk score for prolonged hospitalization, defined as 0-7 days vs. 8+ days of hospital stay, we assigned each patient 1 point for each of the following factors that have been shown to be significantly associated with the risk of prolonged hospitalization: Hb at admission <11.05 g/dL; tumor localization: right-sided colon; fluid intake intraoperatively >3.75 liters; RAA: absent; number of drainage tubes >/=2; postoperative hematocrit level <29.5%. Figure 4 illustrates the relationship between hospital stay and the length of stay (LOS) score.

The analysis reveals that patients who accumulate at least three out of the six identified risk factors are at a significantly higher risk of prolonged hospitalization, defined as a stay exceeding eight days. Specifically, these patients are 5.17 times more likely to experience an extended hospital stay compared to those with fewer than three points. This correlation is not only strong but also highly statistically significant, with a p-value of 0.003.

## Discussion

Our findings highlight key factors that significantly influence both the development of postoperative complications and the length of stay (LOS) after surgery. A complication score was established, grounded in six critical factors linked to adverse postoperative outcomes. Lower hemoglobin levels, indicating anemia, can impair oxygen delivery to tissues, potentially leading to poorer healing and an increased risk of complications. Low hemoglobin levels often require the administration of blood products to improve oxygen delivery and support recovery. However, this intervention carries risks, including allergic reactions, infections, and volume overload. Low serum albumin is a marker of poor nutritional status and has been linked to impaired immune function and increased susceptibility to infections and other postoperative complications. Low albumin levels can compromise tissue integrity, increasing the likelihood of abnormal connections between organs or vessels (fistulas) forming after surgery. Insufficient levels can cause delayed healing, elevate the risk of wound infections, compromise the tissue around the incision, making it more prone to evisceration, and increase the likelihood of eventration. Significant hypoalbuminemia can lead to serious complications, including circulatory collapse due to its effect on oncotic pressure, edema, and anasarca, which increase the risk of additional complications in surgical patients, such as digestive wall edema at high-risk anastomotic sites and the potential for anastomotic leak.

Hypoalbuminemia is common in hospitalized patients and is associated with increased morbidity and mortality. Tumors located in the right side of the colon may be associated with different biological behaviors or surgical challenges that increase the risk of complications. Epidurals are particularly useful for certain procedures because they deliver more effective pain relief than other options, especially during activities such as deep breathing, coughing, or moving in bed. Epidurals have been linked to a decreased risk of post-surgical complications, including conditions like deep vein thrombosis, pulmonary embolism, lung infections, atelectasis, and a reduced need for blood transfusions. Opioid medications, like morphine, though potent, often bring side effects such as nausea, drowsiness, constipation, confusion, reduced gastrointestinal motility, and the potential for dependence on long-term use. Extended periods without oral intake can contribute to nutritional deficits and delayed recovery, which are associated with higher complication rates. Early enteral nutrition after surgery has been associated with lower infection rates and shorter hospital stays. Evidence does not support the notion that bowel rest or fasting contributes positively to surgical recovery or the integrity of anastomoses. Instead, providing nutrition through the gastrointestinal tract can promote better wound healing.

In addition to complications, our analysis identified several factors significantly associated with length of stay (LOS). Excessive fluid administration during surgery can result in fluid overload, which may lead to complications like pulmonary edema or heart strain. Patients experiencing these complications require careful monitoring and management, often resulting in longer hospitalization. RAA minimizes the stress response triggered by surgery. This promotes faster recovery and lessens the likelihood of complications. The absence of RAA necessitates the use of general anesthesia during surgical procedures, which can introduce additional risks for patients. RAA promotes faster recovery by providing superior pain relief, reducing the need for opioids, and minimizing their side effects like nausea and confusion. It allows for earlier mobility and easier participation in physical therapy, which prevents complications and accelerates recovery. By reducing post-surgical risks and enhancing comfort, RAA leads to shorter hospital stays and better overall outcomes for patients. The presence of multiple drainage tubes often indicates a more complicated surgical procedure or the development of complications such as fluid accumulation or abscess formation. These patients require closer monitoring and may experience longer recovery times. Low hematocrit levels post-surgery can signal issues such as bleeding or inadequate fluid resuscitation. Patients with low hematocrit may experience symptoms like fatigue and weakness, necessitating further evaluation and treatment, which can extend hospital stays.

Two key factors identified are tumor localization in the right-sided colon and hemoglobin (Hb) levels below 11.05 g/dL, both of which have been shown to correlate strongly with adverse postoperative outcomes. Their presence in both scoring systems highlights that these variables not only increase the risk of complications but also directly impact the duration of a patient's hospital stay, making them key indicators for overall recovery. Patients with these risk factors tend to require more intensive monitoring, extended recovery times, and additional medical interventions, making them critical indicators for predicting overall patient outcomes and optimizing perioperative care.

At least one-third of patients diagnosed with colorectal cancer (CRC) present with preoperative anemia [21]. Preoperative anemia adversely impacts clinical outcomes for patients, serving as an independent risk factor that heightens the likelihood of postoperative complications. This condition is linked to extended hospital stays and an elevated risk of morbidity and mortality within 30 days following surgery [22]. In a study conducted by Y. Deng et al., the results indicate that preoperative anemia, whether considered alone or alongside postoperative anemia, is linked to a heightened risk of poor overall survival (OS) and disease-free survival (DFS). In contrast, patients without anemia showed the most favorable OS and DFS following curative surgery for colorectal cancer. This highlights preoperative anemia as an independent risk factor for negative long-term outcomes in this group [23].

A study developed by Hartono A et al. introduced the Surabaya scoring system, which demonstrated strong predictive accuracy for postoperative mortality following colorectal cancer surgery, outperforming established models such as AFC, CR-POSSUM, IRCS, and ACS-NSQIP SRC. Key predictors included low albumin levels, elevated pulse rates, dependent functional status, ascites, undergoing a major procedure, dyspnea, and low hemoglobin levels. With a high predictive performance of 0.831, the Surabaya score provides a reliable and effective tool for assessing mortality risk, potentially addressing limitations seen in other scoring systems. This novel model can aid clinicians in better identifying high-risk patients and guiding perioperative management strategies [24].

Another study developed by Kirchhoff P et al. highlights key perioperative complications associated with colorectal surgery and emphasizes the importance of understanding both modifiable and non-modifiable risk factors in reducing patient morbidity and mortality. Advances in laparoscopic techniques, alongside improvements in surgeon training, hospital experience, and standardization of perioperative care, have significantly enhanced patient outcomes and minimized complications. For general surgeons and specialists alike, recognizing these risk factors and employing targeted strategies to prevent, manage, and reduce intra- and postoperative complications is essential for maximizing patient safety and optimizing surgical success [25].

A study by Wallace B et al. identified anastomotic leakage as a critical issue in colorectal surgery, significantly impacting patient morbidity, mortality, and healthcare costs. This review summarized both non-modifiable and modifiable risk factors for leakage, with a detailed analysis of factors across preoperative, intraoperative, and postoperative phases. Among 20 identified factors, three significantly lowered leakage risk: high surgical volume, use of stapling in ileocolic anastomoses, and implementing a diverting ostomy in rectal carcinoma resections. These findings underscore the importance of surgical expertise and specific procedural techniques in minimizing anastomotic leakage, providing actionable insights to enhance surgical outcomes and patient safety in colorectal surgery [26].

In 2016, van Rooijen SJ et al. highlighted that both surgical and non-surgical modifiable risk factors play critical roles in colorectal anastomotic leakage (CAL), a major complication in intestinal surgery with a stable incidence rate of 3-19%. Factors strongly associated with increased CAL risk include diabetes, hyperglycemia, anemia, blood loss, blood transfusions, extended operating times, intraoperative events, contamination, and lack of antibiotic use [27].

A study developed by Collins TC et al. identified several factors associated with prolonged length of stay (LOS) after colorectal surgery, which can help surgeons better explain this outcome to administrators and payers. The study analyzed data from 12,269 patients and found that risk factors for prolonged LOS included male gender, congestive heart failure, weight loss, Crohn’s disease, low preoperative albumin, sepsis, higher ASA class, open surgery, long surgical times, postoperative pneumonia, and complications such as deep venous thrombosis, urinary tract infections, and surgical site infections. These findings provide valuable insights for clinicians to address concerns about prolonged LOS and emphasize the importance of these factors in predicting recovery time after colorectal surgery [28].

Zawadzki M et al. investigated the 30-day reoperation rates following colorectal resections at a single institution, highlighting important risk factors for re-intervention. Out of 464 patients, 11% required reoperation, with the most common causes being anastomotic leakage, postoperative bleeding, and wound dehiscence. Multivariate analysis revealed that patients over 75 years old and those with rectal tumors were at significantly higher risk for reoperation. Furthermore, patients who required re-intervention had longer hospital stays and higher mortality rates. This study emphasizes that reoperation rates in colorectal surgery may be underappreciated, with our findings suggesting that the rate of re-intervention could be as high as 11%, underscoring the importance of monitoring and addressing these complications in colorectal surgical care [29].

A study developed by Kudou K. et al. investigated the risk factors for postoperative complications and hospital mortality following emergency surgery for colorectal perforation. The study found that the most common complications were wound infections and intra-abdominal abscesses. Key risk factors for intra-abdominal abscesses included delayed surgery (greater than 2 days from onset) and a low prognostic nutritional index (PNI<30). A neutrophil-lymphocyte ratio (NLR)<6.15 was identified as a significant risk factor for wound infections. Furthermore, delayed surgery, severe postoperative complications (Clavien-Dindo grade≥IIIa), and a low platelet-lymphocyte ratio (PLR<144) were found to be independent predictors of hospital mortality. These findings underscore the importance of timely intervention and the use of inflammation-based prognostic scores in managing patients with colorectal perforation to improve surgical outcomes and reduce mortality [8].

A study developed by Leung E. et al. highlights the critical need for accurate risk prediction tools in colorectal cancer surgery to better manage postoperative morbidity and mortality, which can vary widely. Traditional metrics like crude mortality rates are insufficient in capturing true outcomes across heterogeneous patient populations. Commonly used scoring systems, such as the Acute Physiology And Chronic Health Evaluation (APACHE) and the American Society of Anesthesiologists (ASA) grades, have limitations due to their complexity or subjectivity. Similarly, while widely used in general surgery, the physiological and operative severity score for the enumeration of mortality and morbidity (POSSUM) and its portsmouth variant (P-POSSUM) tend to overestimate mortality in colorectal patients. In response to these limitations, CR-POSSUM, a more streamlined, colorectal-specific adaptation, has been developed, focusing on fewer, more relevant parameters to improve the accuracy of mortality predictions. This study underlines that standardized, specialty-specific risk assessment models like CR-POSSUM are essential for meaningful outcome comparisons and justifying high-risk surgeries in specialized colorectal units [30].

Our study showed that patients with three or more identified risk factors experienced a complication rate 6.17 times higher, with a highly significant p-value of 0.0008. This highlights the score's strong ability to identify high-risk patients effectively. Also, patients with at least three identified risk factors are 5.17 times more likely to experience prolonged hospitalization (over 8 days) compared to those with fewer than three risk factors, with a statistically significant correlation (p-value of 0.003). The scoring system developed in this study can be an essential tool for healthcare providers to identify at-risk patients, optimize resource allocation, and ultimately enhance patient recovery and outcomes. The integration of the scoring system into routine preoperative assessments has the potential to enhance resource allocation and personalize patient care. While the scoring system identifies significant associations between complications and LOS, the observational design limits the ability to establish causality. Further validation in diverse populations and settings is needed to confirm the scoring system’s generalizability and utility.

## Conclusions

The development of the complication score, based on six key risk factors, demonstrates a robust predictive capability for identifying patients at higher risk of postoperative complications. The significant correlation observed-where patients with three or more points experienced a 6.17-fold increase in complication rates-highlights the importance of monitoring these parameters in clinical settings. With a p-value of 0.0008, the results affirm the score's reliability as a tool for guiding clinical decision-making and resource allocation. The findings from this study underscore the significant impact of various factors on the length of hospital stay following colorectal surgery. The identification of key variables-such as hemoglobin levels, tumor localization, intraoperative fluid intake, locoregional anesthesia use, number of drainage tubes, and postoperative hematocrit levels-highlights their critical role in predicting hospitalization duration. Patients with three or more of these risk factors face a markedly increased likelihood of prolonged stays, specifically being 5.17 times more at risk for hospitalizations exceeding eight days. The strong statistical significance of these results, with a p-value of 0.003, further reinforces the utility of the LOS score in clinical practice.

This scoring system can serve as an essential tool for healthcare providers to identify at-risk patients, optimize resource allocation, and ultimately enhance patient recovery and outcomes. Moreover, the integration of the complication and LOS scores into routine preoperative assessments can facilitate a more personalized care plan, enabling healthcare providers to identify patients who may benefit from closer monitoring and additional support during their recovery. As we move towards more data-driven healthcare practices, these scoring systems offer valuable insights that can lead to improved clinical pathways and ultimately enhance the quality of care for colorectal cancer surgery patients. Continued research and validation of these scores in diverse patient populations will be essential in refining their applicability and effectiveness in various clinical settings.
